# Adverse Event Profile Differences between Trastuzumab Emtansine and Trastuzumab Deruxtecan: A Real-world, Pharmacovigilance Study

**DOI:** 10.7150/jca.86746

**Published:** 2023-10-02

**Authors:** Fen Liu, Guisen Yin, Shuyi Xue, Faisal UL Rehman, Dehua Liao, Yong Pan

**Affiliations:** 1 Department of Pharmacy, Hunan Cancer Hospital, the Affiliated Cancer Hospital of Xiangya School of Medicine, Central South University, Changsha 410011, Hunan, China.; 2 Department of Pharmacy, Yantai Hospital of Traditional Chinese Medicine, Yantai 264000, Shandong, China.; 3 Department of Pharmacy, Affiliated Qingdao Central Hospital of Qingdao University, Qingdao Cancer Hospital, Qingdao, 266042, Shandong, China.; 4 Precision Medicine Center of Oncology, The Affiliated Hospital of Qingdao University, Qingdao University, Qingdao, 266003, China.

**Keywords:** trastuzumab emtansine, trastuzumab deruxtecan, FAERS, pharmacovigilance, real-world, adverse event

## Abstract

**Introduction:** Trastuzumab emtansine(T-DM1) and trastuzumab deruxtecan (T-DXd, formerly DS-8201a), the human epidermal growth factor receptor 2 (HER2)-targeted antibody-drug conjugate (ADC), are commonly used in metastatic breast cancer. However, their real-world safety profile has not been adequately compared.

**Objective:** We aimed to investigate the adverse event (AE) profile of T-DM1 and T-DXd reported by the US Food and Drug Administration Adverse Event Reporting System (FAERS).

**Methods:** All indications were searched for T-DM1 and T-DXd, as primary suspected drugs, from FAERS data (January 2004 to June 2023). Disproportionality analyses were performed by reporting odds ratios (ROR) and proportional reporting ratio (PRR). The odds ratio (OR) of fatal AEs associated with T-DM1 and T-DXd under different exposure factors were performed by univariate and multivariate logistical regression analysis.

**Results:** 3723 and 2045 reports of T-DM1 and T-DXd were submitted to FAERS. Finally, 94 and 61 significant signals for T-DM1 and T-DXd were systematically analyzed. The valid AEs with the highest frequency and the strongest signal intensity for T-DM1 were platelet count decreased (n=108) and hepatopulmonary syndrome (ROR=680.42), respectively. Interstitial lung disease (n=262, ROR=82.55) and pneumonitis (n=89, ROR = 48.34) showed both high frequency and strong signal intensity for T-DXd. The proportion of AEs in each SOC system was different. T-DM1 had a greater proportion of valid AEs in the nervous system, musculoskeletal system, hepatobiliary system, ocular system, cardiac system and hematologic system(*p*<0.05). T-DXd had a greater proportion of valid AEs in the skin disorders, respiratory system, infestations, general system and gastrointestinal system(*p*<0.05). Furthermore, the analysis of fatal AEs in four systems revealed that T-DXd exhibited a significantly higher proportion of fatal outcomes in the hematologic and respiratory system compared to T-DM1. Conversely, T-DM1 had a significantly higher proportion of fatal outcomes in the hepatobiliary system. Neither T-DM1 nor T-DXd exhibited a high mortality ratio in the cardiac system. Logistic regression analysis indicated that advanced age (≥65 years) and male gender were identified as independent risk factors of fatal AEs for both T-DM1 and T-DXd. Additionally, the drug combination therapy, particularly with a CYP3A4 inhibitor, was found to be a risk factor for fatal AEs specifically related to T-DXd.

**Conclusions:** Hematological and respiratory toxicity of T-DXd and hepatobiliary toxicity of T-DM1 exhibited a high incidence of fatal outcomes. It is crucial to identify high-risk factors and enhance the monitoring of AEs during clinical application.

## 1. Introduction

Antibody-drug conjugate (ADC), known as "biological missiles", which contain monoclonal antibodies, cytotoxic drugs and chemical linkers, has emerged as a hotspot for the research and development of antineoplastic drugs. Since the first ADC was approved by the US Food and Drug Administration (FDA) in 2000, 14 ADCs have received market approval worldwide^1^, and 5 of them are approved for solid tumors^2^. Currently, trastuzumab emtansine (T-DM1) and trastuzumab deruxtecan (T-DXd, formerly DS-8201a) are the only two HER2-targeted ADC approved by the FDA in breast cancer.

T-DM1 consists of trastuzumab linked to a cytotoxic microtubule inhibitor through a stable thioether with a drug-antibody ratio (DAR) of 3.5:1. In 2013, FDA licensed T-DM1 for HER2-positive metastatic breast cancer, which was the first approved indication for the treatment of solid malignancy in the class^3^. In 2019, the indication of T-DM1 expanded to the adjuvant therapy of patients with HER2-positive early breast cancer^4^. T-DM1 has revolutionized the treatment of HER2-positive breast cancer but still challenging to escape the fate of drug resistance.

T-DXd, another HER2-targeted ADC, emerges as the times require, comprises trastuzumab, a cleavable tetrapeptide-based linker, and a cytotoxic topoisomerase I inhibitor (DXd). High DAR (approximately 8) and "bystander effect" for T-DXd may contribute to creating continuous glory. In 2019, T-DXd received the FDA approval for the late-line treatment of unresectable or metastatic HER2 positive breast cancer^5^. In 2022, it was updated to second-line treatment and further expanded to include HER-2 low (IHC 1+ or IHC 2+/ISH-) breast cancer patients^6^. In 2021, the FDA approved T-DXd for late-line treatment of adult patients with locally advanced or metastatic HER2-positive gastric or gastroesophageal (GEJ) adenocarcinoma^7^. In 2022, the FDA approved the indication of HER2 mutation unresectable or metastatic non-small cell lung cancer for T-DXd^8^. T-DXd is changing the destiny of HER2-expressing solid tumors^9^.

ADC is a new class of antineoplastic drugs with a special structure different from conventional chemotherapy drugs, which simultaneously achieve high efficiency and low toxicity. The toxicities of ADCs can be regulated by any components, such as targeting antibodies, cytotoxic drugs, linker stability, DAR, bystander effect, site-specific conjugation techniques, etc. Nevertheless, cytotoxic drugs and their metabolites mainly mediate most severe or dose-limited AEs. Structurally, T-DM1 and T-DXd share the same monoclonal antibody. Still, different cytotoxic drugs (microtubule inhibitor vs. topoisomerase I inhibitor), different linkers (stable vs. cleavable), different DAR (3.5 vs. 8), bystander effect (no vs. yes) may predict other toxicity profiles of T-DM1 and T-DXd. The complicated mechanism of ADC makes it difficult to forecast everything exactly, and only extensive experience can provide the proper answer. Recently, the clinical trial of DESTINY-Breast03 10 involved 524 patients has indicated that the incidence of drug-related AEs for T-DXd is higher than that of T-DM1 in HER2-positive metastatic breast cancer, both in any grade and high-grade (≥3 grade). Given the limited number of cases (524 patients), strict eligibility criteria, and limited follow-up time (less than two years), DESTINY-Breast03 cannot fully reflect the safety profile of T-DM1 and T-DXd in the real-world. FAERS, an open pharmacovigilance database that collects worldwide post-marketing safety data submitted to the FDA, compensates for the lack of clinical trials. Therefore, we assess the safety profile of T-DM1 and T-DXd by FAERS to provide a clinical reference for practical and safe application.

## 2. Materials and methods

### 2.1. Data Sources

FAERS is a post-marketing safety monitoring for all FDA-approved drugs. The data used in this study were extracted from FAERS, which were reported spontaneously by consumers, healthcare professsionals and manufacturers from inside or outside the United States. The keywords “trastuzumab emtansine” and “trastuzumab deruxtecan” or brand names “enhertu” and “kadcyla” were utilized to conduct a search in the FAERS database (from January 2004 to June 2023) for all indications, only the reports in which either trastuzumab emtansine or trastuzumab deruxtecan were identified as the primary suspect (PS) drug leading to adverse events were included. To prevent the submission of duplicate reports by both consumers and sponsors, we have excluded reports where "suspect product", "preferred terms", "weight" and "age" were identical. The patient information (age, gender, body weight, reporter and reporter region), drug information (suspect product names, reason for use, concomitant drug), and AE information (preferred terms, seriousness, outcomes and event year) were collected and statistically analyzed.

### 2.2. Data mining

In this paper, the OpenVigil 2 online tool (http://openvigil.Sourceforge.net) was utilized for data mining, the reporting odds ratio (ROR) and proportional reporting ratio (PRR) were used to identify the statistical association between the interested drug and specific AE in the FAERS database, the major algorithms used for signal detection were summarized in Table S1-2. If the criteria listed in Table S2 were met simultaneously, an AE would be considered highly associated with the treatment of the interested drug, with higher values indicating a stronger statistical correlation. Each valid AE was treated as a preferred term (PT) and grouped into the System Organ Class (SOC) based on the Medical Dictionary for Regulatory Activities (MedDRA, version 25.0).

### 2.3. Statistical analysis

We conducted a descriptive analysis of the demographic characteristics, and the chi-square tests or Fisher's exact test in SPSS 26.0 were utilized to analyze the proportion difference of AE reports between T-DM1 and T-DXd. Disproportionality analyses were performed by ROR and PRR, the proportion of valid signals in each SOC was compared through a chi-square test. After removing duplicates, fatal AEs were screened from the original data and classified into the SOC. Univariate and multivariate logistical regression analysis was employed to determine the odds ratio (OR) of fatal AE associated with T-DM1 and T-DXd under different exposure factors, such as sex, age, combination medication, etc.

## 3. Results

### 3.1. Population Characteristics

3723 and 2045 reports of T-DM1 and T-DXd were submitted to FAERS, corresponding to 17319 and 6093 reported AEs, respectively. Understanding that more than one AE was reported in a case makes it easy to see why the actual number of AEs is substantially higher than the number of reports. The population characteristics are presented in Table 1. Healthcare professionals reported most AEs, 77.36% for T-DM1 and 87.78% for T-DXd. The reports of T-DXd were primarily submitted from the Americas (49.63%) and Japan (25.48%), while the reports of T-DM1 were dispersedly submitted, primarily from the Americas (29.65%) and Canada (11.74%). T-DM1 was used mainly in breast carcinoma (69.78%). While T-DXd was primarily used in breast carcinoma (59.66%), gastric carcinoma (15.70%), and lung carcinoma (4.11%). As a result, the proportion of male patients in T-DXd (18.44%) was nearly eight times higher than that of T-DM1 (2.39%).

### 3.2. Disproportionality Analyses for T-DM1 and T-DXd

**Figure 1:**
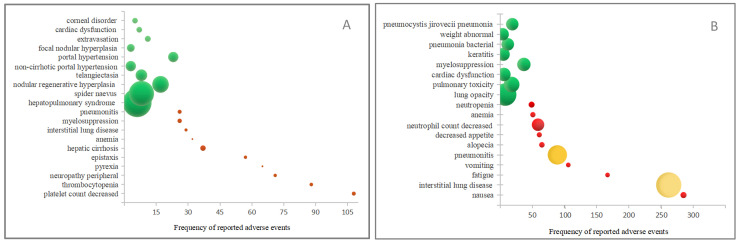
The top ten adverse events (AEs) with the highest frequency and strongest signal intensity for (A) T-DM1 and (B) T-DXd. The AE frequency is represented by the x-axis, the various AEs are represented by the y-axis, and the bubble size reflects the ROR value. The top ten AEs with the highest frequency are shown in red bubbles, the top ten AEs with the highest signal strength are shown in green bubbles, and AEs that display both high frequency and strong intensity are shown in yellow bubbles.

Excluding the AE obviously unrelated to the drug, T-DM1 and T-DXd had 94 and 61 significant signals separately. The top ten AEs with the highest frequency and strongest signal intensity for T-DM1 and T-DXd were analyzed. As shown in Figure 1A and Table S3-4, the AE with the highest frequency for T-DM1 was platelet count decreased(n=108), followed by thrombocytopenia (n=88) and peripheral neuropathy (n=71). The AE with the strongest signal intensity for T-DM1 were hepatopulmonary syndrome (ROR=680.42), spider naevus (ROR=522.14) and nodular regenerative hyperplasia (ROR=225.09). Likewise, as shown in Figure 1B and Table S3-4, interstitial lung disease (n=262, ROR=82.55) and pneumonitis (n=89, ROR=48.34) showed both high frequency and strong signal intensity for T-DXd.

**Figure 2:**
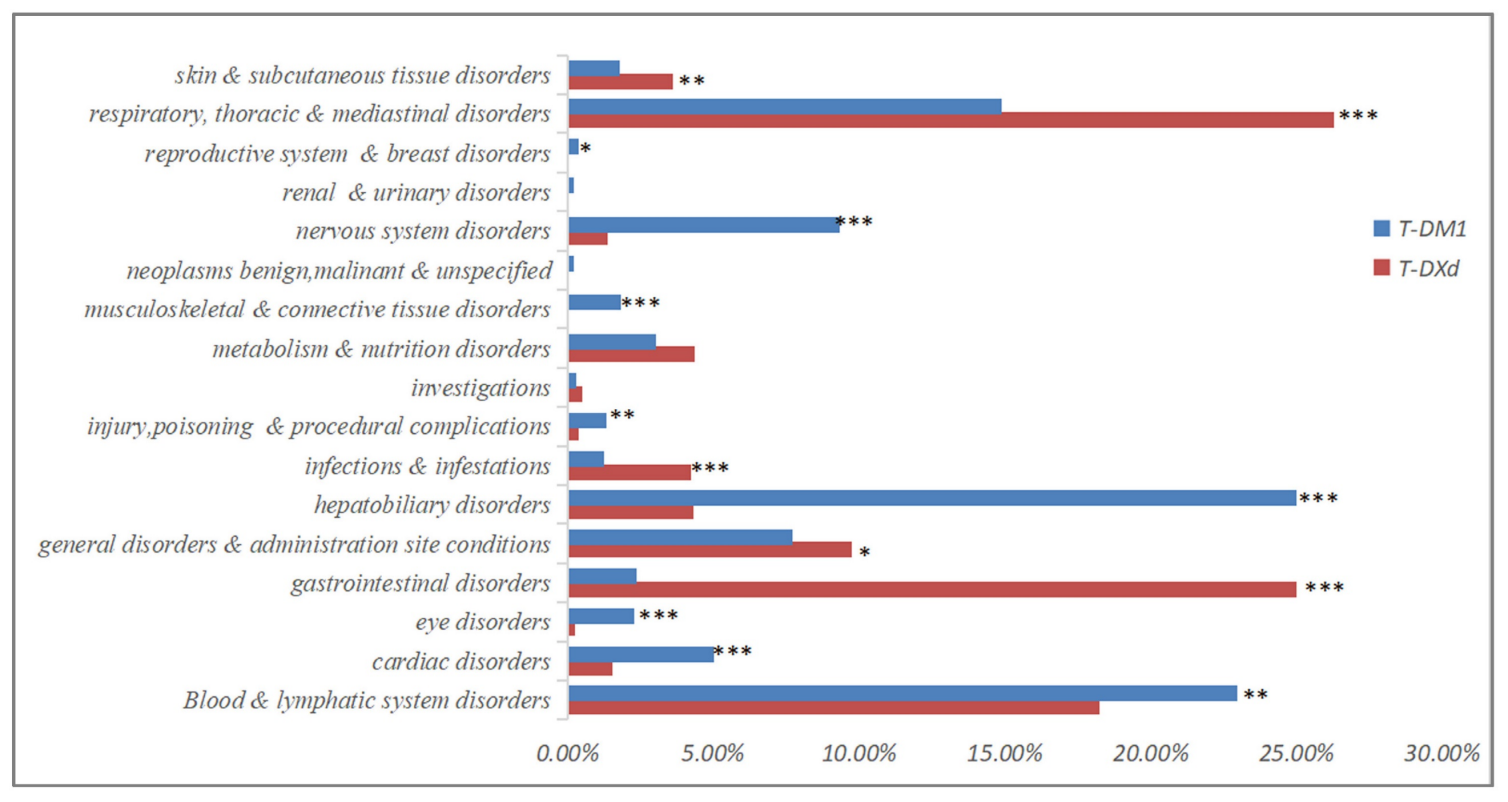
The proportion of T-DM1 and T-DXd-related significant Adverse Events (AEs) at the System Organ Class (SOC) level. *: *p*<0.05; **: *p*<0.01; ***: *p*<0.001.

To further analyze the differences between T-DM1 and T-DXd, the entire analysis results of signal mining at the System Organ Class (SOC) level were presented in Figure 2 and Table S5. As shown in Figure 2, the proportion of AEs in each SOC system was different. T-DM1 exhibited a greater proportion of AEs in the nervous system, musculoskeletal system, hepatobiliary system, ocular system, cardiac system and hematological system (*p<*0.05). T-DXd exhibited a greater proportion of AEs in the skin disorders, respiratory system, infestations, general systems and gastrointestinal system (*p<*0.05).

Although within the same category, there were notable differences in AEs between T-DM1 and T-DXd. As indicated in Table S5, T-DM1 was associated with a greater proportion of thrombocytopenia-related AEs, including thrombocytopenia (n=88) and platelet count decreased (n=108). T-DXd was linked to more neutropenia-related AEs, such as febrile neutropenia(n=35), neutropenia(n=49) and neutrophil count decreased(n=59) in the hematological system. In the metabolism and nutrition disorders, T-DM1 was associated with more electrolyte imbalance-related AEs, T-DXd suffered more AEs with decreased appetite. In the nervous system, T-DM1 suffered more AEs associated with peripheral sensory neuropathy, T-DXd hardly developed peripheral neuropathy. In skin and subcutaneous tissue disorders, T-DM1 suffered more AEs associated with skin toxicity, such as spider naevus and telangiectasia, T-DXd suffered more alopecia.

Although T-DM1 and T-DXd may have overlapping AEs, statistical analysis was conducted to determine the differential proportions of each AE. As shown in Table S5, in the hematological system, T-DM1 exhibited a significantly higher proportion of AEs related to cytopenia, thrombocytopenia, and decreased platelet count compared to T-DXd (*p*<0.05). Similarly, in the hepatobiliary system, T-DM1 showed a greater proportion of AEs associated with increased blood bilirubin levels, hepatotoxicity, abnormal hepatic function, liver disorder and jaundice (*p*<0.05). Additionally, in the nervous system, brain edema and cerebral hemorrhage were more frequently reported in T-DM1 than in T-DXd (*p*<0.05). Conversely, in the respiratory system, T-DXd demonstrated a significantly higher proportion of AEs related to pneumonitis and interstitial lung disease compared to T-DM1 (*p*<0.05), although T-DM1 exhibited a significantly higher proportion of AEs related to pulmonary fibrosis compared to T-DXd (*p*<0.05).

### 3.3. Fatal AEs linked to T-DM1 and T-DXd

**Figure 3:**
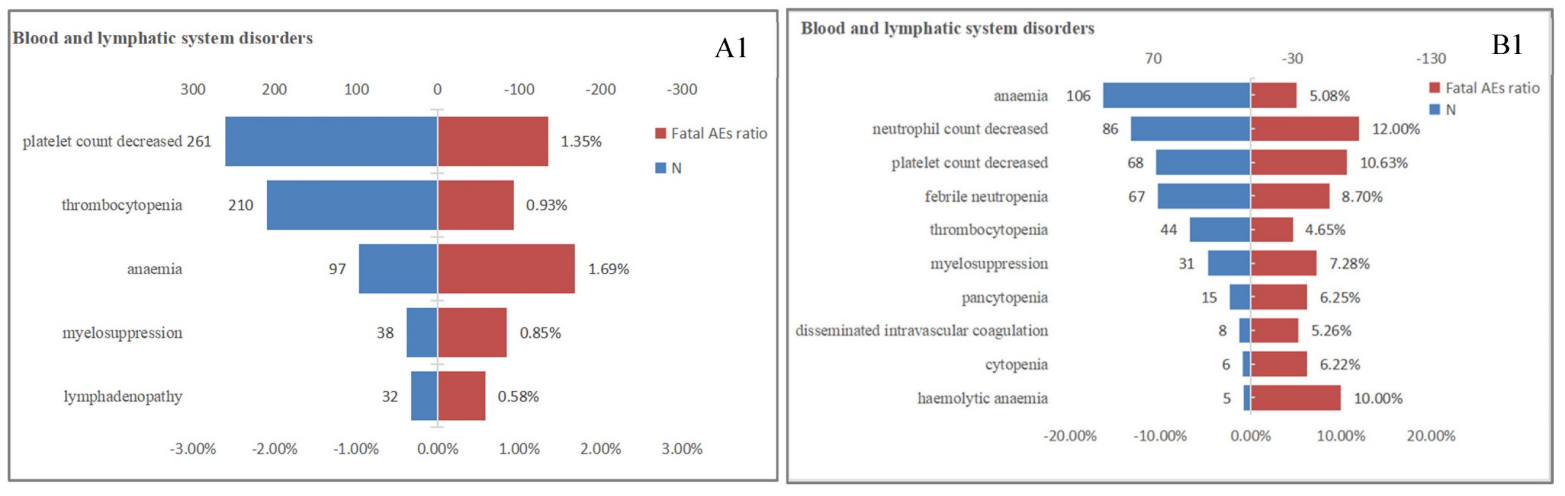

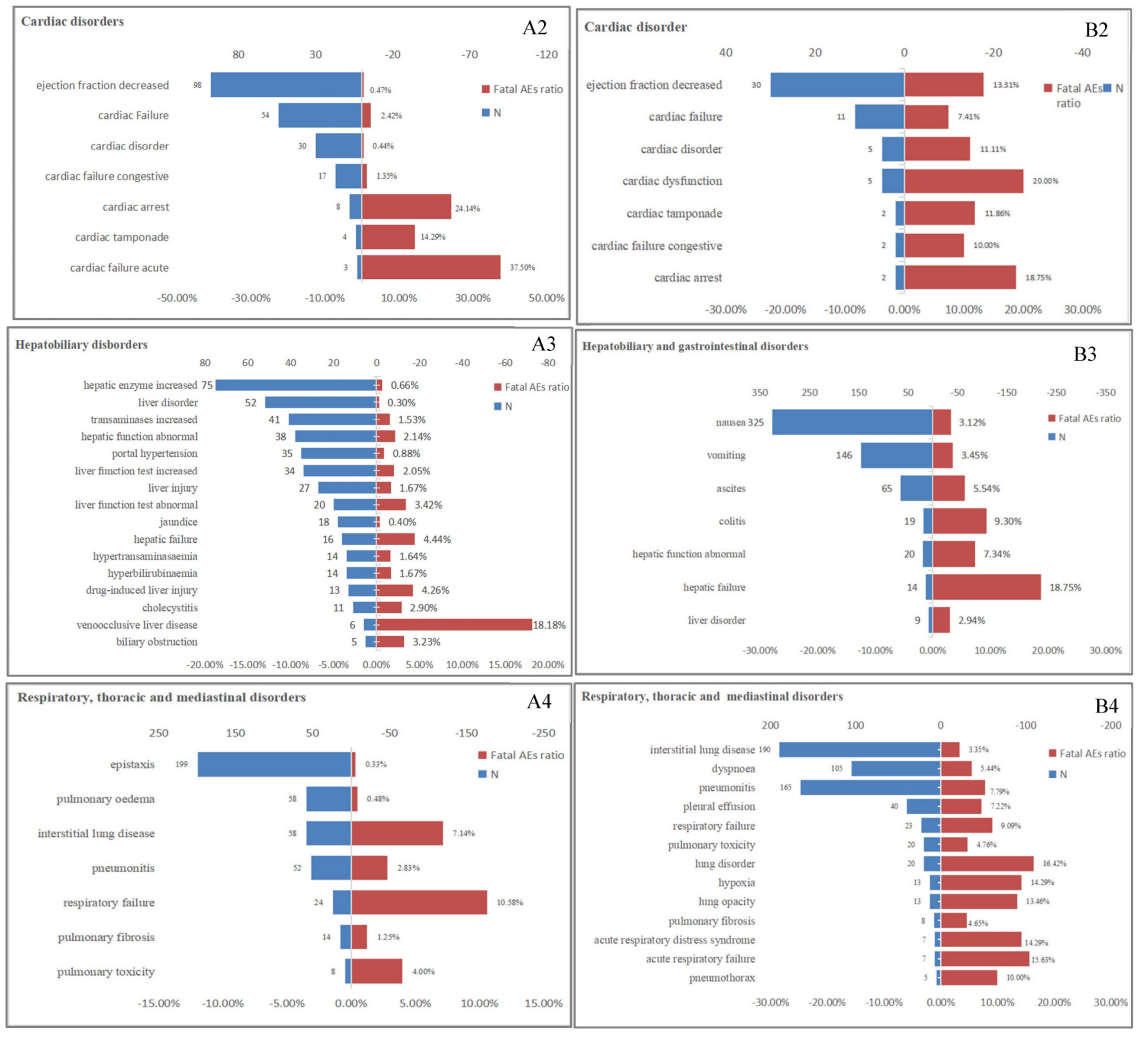
The proportion of fatal AEs in each System Organ Class (SOC) associated with (A) T-DM1 and (B) T-DXd. AEs, adverse effects; N, the number of T-DM1/T-DXd -associated AEs.

Lethal AEs were significant factors limiting the widespread use of drugs. Table 1 indicated that the proportion of serious outcomes in T-DM1 (81.82%) was significantly higher than T-DXd(68.85%), while the proportion of fatal outcomes with T-DXd (23.57%) was nearly twice as high as T-DM1 (12.60%). Our previous research findings have exhibited a high proportion of AEs in multiple systems, including the hematologic, cardiac, respiratory and hepatobiliary systems for both T-DM1 and T-DXd. Furthermore, we conducted an additional analysis to determine the proportion of fatal AEs associated with four SOCs (Figure 3). In the hematologic system, T-DXd exhibited a significantly higher mortality ratio compared to T-DM1. The most prevalent fatal AEs for T-DM1 was anemia (1.69%), while neutrophil count decreased (12.00%) was the leading cause of death for T-DXd. Neither T-DM1 nor T-DXd exhibited a high mortality ratio in the cardiac system. It was possible that the cardiotoxicity fatality ratio may be overestimated due to the low incidence of cardiotoxicity. In the hepatobiliary system, the mortality ratio associated with T-DM1 was overwhelmingly higher than that of T-DXd. Oppositely, in the respiratory system, the mortality ratio associated with T-DXd was overwhelmingly higher than that of T-DM1, The most prevalent fatal AEs for T-DM1 and T-DXd respectively were respiratory failure (10.58%) and lung disorder (16.42%).

Further, univariate and multivariate logistical regression analyses were employed to determine the odds ratio (OR) of fatal AE associated with T-DM1 and T-DXd under different exposure factors. The results presented in Table 2-3 indicated that age, gender, and drug combination were independent factors that significantly influence fatal AEs associated with T-DM1 and T-DXd. As shown in Table 2, compared with patients under the age of 65, the risk of fatal AEs associated with T-DM1 was 2.65 times higher in patients aged 65-74 years and 31.52 times higher in patients aged 75 years or older. Additionally, females had a lower risk of fatal AEs. Combination drugs other than CYP3A4 inhibitors were associated with a lower risk of fatal AEs compared to non-combination therapy. Similarly, as indicated in Table 3, compared with patients under the age of 65, the risk of fatal AEs associated with T-DXd was two times higher in patients aged 65-74 years and those over the age of 75. Females exhibited a lower risk of fatal AEs. Interestingly, the risk of fatal AEs associated with T-DXd in combination with CYP3A4 inhibitors was 2.13-fold higher than non-combination therapy (OR=2.130[1.390,3.264],* p*=0.001), which was a greater risk than when combination with other drugs (OR=1.343[1.054,1.711],* p*=0.017).

## 4. Discussion

### 4.1. Hematotoxicity

Hematotoxicity is the most common dose-limited toxicity of cytotoxic drugs, and ADCs are no exception. The most frequent hematological toxicity for T-DM1 is thrombocytopenia, with an incidence from 21.0% to 52.9%^11^-^13^. Likewise, platelet count decreased(n=108) and thrombocytopenia (n=88) were the top 2 AEs with the highest frequency for T-DM1 in our study. Furthermore, thrombocytopenia is also the most frequent (5.7% to 24.9%) high-grade (≥3 grade) AEs in clinical trials^11^-^13^. Fortunately, high-grade (≥3 grade) thrombocytopenia does not increase the frequency of high-grade (≥3 grade) hemorrhage, most patients return the platelet count to the lower limit of treatment before the next cycle^14^.

Interestingly, compared with non-Asian patients, Asian patients are associated with a significantly higher frequency of high-grade (≥3 grade) thrombocytopenia (21.6% vs. 5.7%) for T-DM1 treatment^15^. While the mechanism is still unclear. In vitro experiments showed that T-DM1 did not directly inhibit platelet activation and aggregation but inhibited differentiation of megakaryocytes (MKs)^16^. T-DM1 was internalized into MKs not by HER-2, at least partially by FcγRIIa, then released the DM1 to damage the microtubule structure of MKs. However, another study did not entirely agree with it^17^. Regardless of the absence of FcγRIIa in mice and HER-2 receptor in MKs and platelets, T-DM1 still permeated MKs and platelets to inhibit differentiation of MKs and delay production of proplatelet, neither depended on FcγRIIa nor HER2.

Many "blockbuster" clinical studies^10^, ^18^, ^19^ have confirmed that the hematological toxicity profile of T-DXd is much different from T-DM1. The incidence of neutropenia (33.2% to 42.8%), anemia (29.9% to 33.2%), thrombocytopenia (21.2% to 24.9%) and leukopenia (21.2% to 30.0%) for T-DXd was similar. While the incidence of high-grade (≥3 grade) neutropenia (13.7% to 20.7%) is significantly higher than others (4.3% to 8.7%) for T-DXd. Likewise, our research indicated that the most frequency AE of T-DXd in hematotoxicity was associated with neutropenia, including febrile neutropenia (n=35), neutropenia (n=49), neutrophil count decreased (n=59) (Table S5), neutrophil count decreased was also the AE with the highest fatal ratio in hematotoxicity (Figure 3). To sum up, hematotoxicity is the most common dose-limited toxicity for both T-DM1 and T-DXd, which can be safely managed by timely monitoring, appropriate dosing adjustment, and supporting therapy^20^.

### 4.2. Respiratory toxicity

Interstitial lung disease (ILD) or pneumonitis were potentially life-threatening AEs for T-DXd, which were warned by the FDA in a "black box" and suggested permanently stopping for patients with grade≥2 ILD or pneumonitis. In our research, ILD (n=262, ROR=82.55) and pneumonitis (n=89, ROR=48.34) showed both high frequency and strong signal intensity for T-DXd (Figure 1B).T-DM1 also exhibited a strong signal for ILD (n=29, ROR=7.43) and pneumonitis (n=26, ROR=12.68), but the occurrence ratio was significantly lower than that of T-DXd (*p*<0.05) (Table S5). Further, our research demonstrated that T-DXd had a greater proportion of both all outcomes AEs and fatal AEs in the respiratory system than that of T-DM1. A meta-analysis showed that the incidence of all-grade and grade≥3 pneumonitis associated T-DXd respectively were 13.58 % and 2.19%, which were higher than T-DM1^21^. The fundamental processes causing ADC-related lung injury may include early payload withdrawal due to target-dependent or target-independent uptake, bystander effect, or circulating payload^22^. The pathogenesis of ADC-associated ILD remains elusive, and the management experience is still sufficient. It is strongly recommended to maintain a high level of vigilance for ILD during clinical practice and promptly identify individuals at elevated risk^23^.

Our research discovered that the highest frequency and strongest signal for T-DM1 in the respiratory system respectively were epistaxis (n=57, ROR=8.77) and hepatopulmonary syndrome (n=6, ROR=680.42). Epistaxis was the most common AE for T-DM1, with a frequency of 21.5% to 25.0% for any grade and less than 1% for high grade (≥3)^12^, ^13^. Based on the limited evidence, epistaxis may be related to DM1-induced telangiectasia, mainly manifested as mucosal bleeding^15^, which may cause minor bleeding such as epistaxis, gingival bleeding, and spider nevus but can also cause severe gastrointestinal bleeding, diffuse mucocutaneous telangiectasias^24^, ^25^. We recommend screening for bleeding risk in patients with mucosal or cutaneous telangiectasia. Hepatopulmonary syndrome is a pulmonary complication of portal hypertension or liver cirrhosis, which is first documented in our research and may be related to the hepatotoxicity caused by T-DM1.

### 4.3. Digestive Toxicity

Our research showed that nausea (n=285, ROR=4.75) was the most common AE for T-DXd in gastrointestinal systems, followed by vomiting (n=106, ROR=2.82). T-DXd occurred more frequently in gastrointestinal systems than T-DM1(*p*<0.001), consistent with DESTINY-Breast03^10^. Both the American Society of Clinical Oncology (ASCO)^26^ and National Comprehensive Cancer Network (NCCN)^27^ guidelines considered T-DM1 and T-DXd to be low and moderate emetogenic potential respectively, recommend prophylactic antiemetic therapy for T-DXd with a combination of 5-HT3 receptor antagonist and dexamethasone, add NK1 blockers when necessary. No routine prophylaxis was recommended for T-DM1. The analysis of combination data in our study indicated that clinical practice followed a similar approach.

Hepatotoxicity is common dose-limited toxicity for T-DM1, liver failure, and even death have been reported in T-DM1 treatment. Our research indicated that T-DM1 exhibited an overwhelmingly higher ratio of both all outcomes and fatal outcomes AE than that of T-DXd in the hepatobiliary system. The highest frequency and strongest signal for T-DM1 associated with hepatotoxicity was hepatic cirrhosis (n=37, ROR=23.58) and nodular regenerative hyperplasia (NRH) (n=17, ROR=225.09). It can be seen that T-DM1-induced hepatotoxicity is not limited to laboratory abnormalities, but also manifested as the decline of organ function, such as portal hypertension, cirrhosis, NRH, etc. Hepatic cirrhosis was not mentioned on the label, with the ratio much higher than we expected. NRH is a rare but very serious AE. It is recommended to permanently terminate T-DM1 treatment once it happens. Monitoring hepatic function is strongly recommended prior to initiation and each dose. Despite the HER2-dependent pathway playing a key role in the mechanism of hepatotoxicity for T-DM1, an HER-2-independent mechanism that T-DM1 interacts with CKAP5 on the cell surface of hepatocytes by DM1 and damages the plasma membrane, leading to calcium influx and microtubule network disorder, may partly explain the Off-target effect of T-DM1^28^.

### 4.4. Cardiotoxicity

The cardiotoxicity of trastuzumab has been widely regarded as concerning, especially in combination with anthracyclines, with a 16% incidence of III or worse cardiac dysfunction^29^. Despite containing trastuzumab, the incidence of III or worse cardiac dysfunction associated with T-DM1 was less than 1%, which was significantly lower than that of trastuzumab^30^, and T-DXd was the same^18^. Similarly, our research indicated that the proportion of AEs with all outcomes and fatal outcomes in the cardiac system was not high in both T-DM1 and T-DXd. However, when compared to T-DXd, T-DM1 showed a higher proportion of AEs than T-DXd (*p*<0.001). Despite this low rate of cardiotoxicity, both T-DM1 and T-DXd are recommended for baseline cardiac assessment and appropriate monitoring.

### 4.5. Others

T-DM1 contains a potent microtubule inhibitor. Even though smart structure design improves targeting, it still increases the risk of peripheral neurotoxicity^31^. While, compared to taxane, T-DM1 showed a much lower relative risk of any grade peripheral neuropathy and peripheral sensory neuropathy^32^. In our research, neuropathy peripheral (n=71, ROR=8.97) showed the most frequency for T-DM1 in the nervous system. Besides, central neuropathy, such as cerebral hemorrhage (n=6, ROR=8.65) and brain edema (n=10, ROR=8.74), was discovered in our research, which were rare but severe and not mentioned in the instruction. Sporadic Cases have reported cerebellar hematoma^33^ and brain edema^34^ occurred during brain radiotherapy combined with T-DM1, and the recurrence of brain metastases has been excluded, suggesting that there may be an interaction between radiotherapy and T-DM1, special attention should be paid to it.

Ocular toxicity is a rare drug-related AE that significantly affects the quality of life. Dry eye (n=16, ROR=4.53) was the most common ocular AE for T-DM1. The corneal disease (n=5, ROR=24.34) and blindness (n=10, ROR=2.47) for T-DM1 and keratitis (n=5, ROR=21.95) for T-DXd were not mentioned in the instruction. A cross-sectional investigation showed that T-DM1-related corneal illness usually showed up as low-grade, mid-peripheral corneal epithelial lesions that were reversible, mostly asymptomatic^35^. Still, the AE of blindness served as a warning that ocular toxicity should not be underestimated.

In vitro, both DM1 and DXd are primarily metabolized by CYP3A4. Compared to monotherapy, combined with itraconazole, a strong CYP3A inhibitor, increased the steady state AUC_17d_ by 11% for T-DXd and 18% for DXd, but did not impact the safety profile of T-DXd^36^. No drug-drug interaction studies with T-DM1 have been conducted. While, the logistical regression analysis in our study revealed that the combination with CYP3A4 inhibitors increased the risk of fatal AEs associated T-DXd, but not T-DM1. Compared to T-DM1, T-DXd was associated with a higher frequency of co-administration with CYP3A4 inhibitors, such as aprepitant, clarithromycin, fluconazole and ciprofloxacin, etc. The available data on drug interactions remains limited, and our study serves as a reminder that the potential impact of combination drugs, particularly combined with CYP3A4 inhibitors, should not be overlooked in clinical practice.

## 5. Limitations

Our study has the following significant shortcomings: (1) Spontaneous reports in the FAERS database may be subject to quantitative bias and incomplete reporting; (2) The reports are mainly from America and Europe, with few data from Asians or Africans; (3) ROR only indicates the increased risk of reported AE and does not reflect the actual clinical risk; (4) Due to the lack of exposure data, we could not calculate morbidity and mortality, (5) T-DXd come to market relatively late, insufficient exposure of T-DXd may lead to a bias in the comparison of the differences with T-DM1.

## 6. Conclusions

In our study, 3723 and 2045 reports of T-DM1 and T-DXd were submitted to FAERS up to June 2023. Finally, a systematic analysis and comparison of 94 and 61 significant signals for T-DM1 and T-DXd were performed.Additionally, we examined the effects of various exposure parameters on fatal AEs and contrasted the proportion of fatal AEs between T-DM1 and T-DXd across the four systems. Our research indicated that the toxicity spectra of T-DM1 and T-DXd are typical and mostly in line with the results of the DESTINY-Breast03 Clinical Trials. The extensive real-world data afforded us an opportunity to investigate the fatal AEs, as well as rare AEs that had a high frequency or strong ROR in our study but were not mentioned in clinical findings or instruction. This provided a better illustration of what an actual pharmacovigilance research entails. In conclusion, there are notable differences between the toxicity profiles of T-DM1 and T-DXd, with complex pathways controlled by a variety of variables. As a result, safety management standards are urgently needed to effectively guide clinical procedures employing ADCs.

## Supplementary Material

Supplementary tables.

## Figures and Tables

**Table 1:** Clinical characteristics of patients treated with T-DM1 and T-DXd in the FAERS database.

Characteristics		Reports, N (%)		*p*-value
		T-DM1	T-DXd	
Age	<18	62 (1.67)	73 (3.57)	**<0.001^b^**
	18~65	1569 (42.14)	533 (26.06)	
	>65	376 (10.10)	366 (17.90)	
	Not Specified	1716 (46.09)	1073 (52.47)	
Gender	Male	89 (2.39)	377 (18.44)	**<0.001^b^**
	Female	3190 (85.68)	1288 (62.98)	
	Not Specified	444 (11.93)	380 (18.58)	
Reporter	Consumer	840 (22.56)	248 (12.13)	**<0.001^a^**
	Healthcare Professional	2880 (77.36)	1795 (87.78)	
	Not Specified	3 (0.08)	2 (0.10)	
Reporting region (TOP 6)	Americas	1104 (29.65)	1015 (49.63)	**<0.001^a^**
	Canada	437 (11.74)	53 (2.59)	
	Japan	320 (8.60)	521 (25.48)	
	Germany	157 (4.22)	/	
	France	137 (3.68)	114 (5.57)	
	Britain	132 (3.55)	39 (1.91)	
	Italy	/	50 (2.44)	
Seriousness	Non-Serious	677 (18.18)	637 (31.15)	**<0.001^b^**
	Serious	3046 (81.82)	1408 (68.85)	
Outcome	Died	469 (12.60)	482 (23.57)	**<0.001^b^**
	Life Threatening	34 (0.91)	18 (0.88)	
	Disabled	51 (1.37)	12 (0.59)	
	Hospitalized	631 (16.95)	492 (24.06)	
Indication (TOP 3)	Breast carcinoma	2598 (69.78)	1220 (59.66)	**<0.001^a^**
	Gastric carcinoma	/	321 (15.70)	
	Lung carcinoma	15 (0.40)	84 (4.11)	
	Salivary Gland Cancer	17 (0.46)		
Event year	2013	71 (1.91)	/	**<0.001^a^**
	2014	231 (6.20)	/	
	2015	545 (14.64)	/	
	2016	334 (8.97)	/	
	2017	298 (8.00)	/	
	2018	316 (8.49)	/	
	2019	302 (8.11)	/	
	2020	438 (11.76)	240 (11.74)	
	2021	524 (14.07)	416 (20.34)	
	2022	507 (13.62)	893 (43.67)	
	2023	155 (4.16)	496 (24.25)	

a: Fisher's exact test; b: Chi-square test. Statistically significant values are marked in boldface.

**Table 2:** Univariate and multivariate logistic regression analyses of the odds ratio for T-DM1-related fatal adverse events

Variable	Factor	Univariate analysis		Multivariate analysis	
		*p*	OR (95%CI)	*p*	OR (95%CI)
Age	<65 (Reference)	/	1	/	1
	65-74	**<0.001**	**2.631 (1.875,3.692)**	**<0.001**	**2.651 (1.887,3.726)**
	≥75	**<0.001**	**32.250 (14.111,73.703)**	**<0.001**	**31.520 (13.737,72.323)**
Gender	Male (Reference)	/	1	/	1
	Female	**0.001**	**0.424 (0.261,0.290)**	**0.001**	**0.424 (0.254,0.707)**
Off-label use	No (Reference)	/	1	/	/
	Yes	0.921	0.976(0.605,1.575)	/	/
Concomitant drug	No (Reference)	/	1	/	1
	CYP3A4 inhibitor	0.706	1.230 (0.420,3.608)	0.679	1.258 (0.424,3.733)
	Others	**0.046**	**0.810 (0.659,0.996)**	**0.015**	**0.765 (0.617,0.949)**

OR: Odds Ratio; I: Confidential Interval. Statistically significant values are marked in boldface.

**Table 3:** Univariate and multivariate logistic regression analyses of the odds ratio for T-DXd-related fatal adverse events.

Variable	Factor	Univariate analysis		Multivariate analysis	
		*p*	OR (95%CI)	*p*	OR (95%CI)
Age	<65(Reference)	/	1	/	1
	65-74	**<0.001**	**2.822 (1.601,4.973)**	**0.003**	**2.462 (1.369,4.429)**
	≥75	**0.004**	**2.641 (1.376,5.070)**	**0.011**	**2.377 (1.218,4.640)**
Gender	Male (Reference)	/	1	/	1
	Female	**<0.001**	**0.427 (0.335,0.545)**	**<0.001**	**0.447 (0.348,0.572)**
Off-label use	No (Reference)	/	1	/	/
	Yes	0.629	1.084 (0.782,1.502)	/	/
Concomitant drug	No (Reference)	/	1	/	1
	CYP3A4 inhibitor	**<0.001**	**2.317 (1.534,3.501)**	**0.001**	**2.130 (1.390,3.264)**
	Others	0.073	1.551 (1.229,1.958)	**0.017**	**1.343 (1.054,1.711)**

OR: Odds Ratio; CI: Confidential Interval. Statistically significant values are marked in boldface
