# Supplementary material for: Adverse Event Profile Differences between Trastuzumab Emtansine and Trastuzumab Deruxtecan: A Real-world, Pharmacovigilance Study
Source: J Cancer. 2023 Oct 2;14(17):3275–84. doi: 10.7150/jca.86746 (PMC10622999; doi:10.7150/jca.86746)
Supplement: Supplementary file 1 — Supplementary tables. [file jcav14p3275s1.pdf]

supplementary materials：

Table S1 Two-by-two contingency table for disproportionality analyses.

	Adverse events of interest	All other adverse events of interest	total
Drug of interest	a	b	a+b
All other drugs of interest	c	d	c+d
Total	a+c	b+d	a+b+c+d

Table S2 Summary of major algorithms used for signal detection

Algorithms	Equation	criteria
ROR	ROR=ad/bc<br>95% CI=$e^{\ln(ROR)\pm 1.96（1/a+1/b+1/c+1/d）\text{^}0.5}$	95% CI＞1，N⩾3
PRR	PRR=(a/(a+c))/(b/(b+d))<br>χ^2=［(ad-bc)^2］(a+b+c+d)/［(a+b)(c+d)(a+c)(b+d)］	PRR⩾2，χ^2⩾4，N⩾3

Abbreviations: ROR, reporting odds ratio;N, the number of reports AEs;CI: confidence interval; PRR, proportional reporting ratio; χ^2, chi-squared;

Table S3 The top ten adverse events(AEs) with the highest frequency for T-DM1 and T-DXd

Drug	PT	SOC	Freq(%)	ROR(95% CI)
T-DM1	platelet count decreased	investigations	108(7.91)	12.46(10.26,15.11)
	thrombocytopenia	blood and lymphatic system disorders	88(6.44)	9.89(7.99,12.24)
	neuropathy peripheral	nervous system disorders	71(5.20)	8.97(7.08,11.37)
	pyrexia	general disorders and administration site conditions	65(4.76)	2.34(1.83,2.99)
	epistaxis	respiratory, thoracic and mediastinal disorders	57(4.17)	8.77(6.74,11.41)
	hepatic cirrhosis*	hepatobiliary disborders	37(2.71)	23.58(17.02,32.66)
	anemia	blood and lymphatic system disorders	32(2.34)	2.08(1.46,2.94)
	interstitial lung disease	respiratory, thoracic and mediastinal disorders	29(2.12)	7.43(5.15,10.72)
	myelosuppression	blood and lymphatic system disorders	26(1.90)	15.13(10.27,22.29)
	pneumonitis	respiratory, thoracic and mediastinal disorders	26(1.90)	12.68(8.61,18.68)
T-DXd	nausea	gastrointestinal disorders	285(15.85)	4.75(4.19,5.39)
	interstitial lung disease	respiratory, thoracic and mediastinal disorders	262(14.57)	82.55(72.44,94.07)
	fatigue	general disorders and administration site conditions	167(9.29)	2.79(2.38,3.26)
	vomiting	gastrointestinal disorders	106(5.90)	2.82(2.32,3.43)
	pneumonitis	respiratory, thoracic and mediastinal disorders	89(4.95)	48.34(39.05,59.84)
	alopecia	skin and subcutaneous tissue disorders	65(3.62)	3.89(3.04,4.98)
	decreased appetite	metabolism and nutrition disorders	61(3.39)	3.34(2.59,4.31)
	neutrophil count decreased	investigations	59(3.28)	20.12(15.52,26.08)
	anemia	blood and lymphatic system disorders	51(2.84)	3.58(2.71,4.73)
	neutropenia	blood and lymphatic system disorders	49(2.73)	4.59(3.46,6.10)

*not mentioned in the instruction;

Abbreviations: SOCs: System Organ Classes;N, the number of reports AEs; PTs: Preferred Terms; ROR, reporting odds ratio; CI: confidence interval

Table S4 The top ten AEs with the strongest signal intensity for T-DM1 and T-DXd

Drug	PT	SOC	Freq(%)	ROR(95% CI)
T-DM1	hepatopulmonary syndrome*	respiratory, thoracic and mediastinal disorders	6(0.44)	680.42(290.29,1594.81)
	spider naevus*	skin and subcutaneous tissue disorders	8(0.59)	522.14(252.12,1081.34)
	nodular regenerative hyperplasia	hepatobiliary disorders	17(1.24)	225.09 (138.27 ,366.44)
	telangiectasia*	skin and subcutaneous tissue disorders	8(0.59)	110.21 (54.65,222.28)
	non-cirrhotic portal hypertension	hepatobiliary disorders	3(0.22)	86.34 (27.57,270.43)
	portal hypertension	hepatobiliary disorders	23(1.68)	85.34 (56.40,129.13)
	focal nodular hyperplasia	neoplasms benign,malinant and unspecified	3(0.22)	48.38 (15.51, 150.92)
	extravasation	general disorders and administration site conditions	11(0.81)	27.83 (15.37,50.42)
	cardiac dysfunction	cardiac disorders	7(0.51)	25.49 (12.12,53.63)
	corneal disorder*	eye disorders	5(0.37)	24.34 (10.10,58.66)
T-DXd	interstitial lung disease	respiratory, thoracic and mediastinal disorders	262(14.57)	82.55 (72.44,94.07)
	lung opacity*	respiratory, thoracic and mediastinal disorders	8(0.44)	65.04 (32.35,130.75)
	pneumonitis	injury,poisoning and procedural complications	89(4.95)	48.34 (39.05,59.84)
	pulmonary toxicity	respiratory, thoracic and mediastinal disorders	18(1.00)	31.83 (19.99,50.69)
	cardiac dysfunction	cardiac disorders	6(0.33)	23.38 (10.47,52.18)
	myelosuppression	blood and lymphatic system disorders	37(2.06)	23.24 (16.78,32.19)
	keratitis*	eye disorders	5(0.28)	21.95 (9.11,52.89)
	pneumonia bacterial*	infections and infestations	12(0.67)	20.45 (11.58,36.11)
	weight abnormal	investigations	4(0.22)	20.10 (7.52,53.69)
	pneumocystis jirovecii pneumonia*	infections and infestations	19(1.06)	20.03 (12.74,31.49)

*Not mentioned in the instruction;

Abbreviations: SOCs: System Organ Classes;N, the number of reports AEs; PTs: Preferred Terms; ROR, reporting odds ratio; CI: confidence interval

Table S5 The entire analysis results of signal mining for T-DM1 and T-DXd

SOC system	PT	T-DM1			T-DXd			*p* values
		N	ROR (95% CI)	PRR(χ^2)	N	ROR (95% CI)	PRR(χ^2)	
blood and lymphatic system disorders	myelosuppression	26	15.13(10.27,22.29)	14.96(324.20)	37	23.24(16.78,32.19)	22.83(748.08)	0.237[b]
	splenomegaly*	15	14.51(8.73,24.13)	14.42(173.73)	/	/	/	/
	cytopenia	10	11.15(5.99,20.77)	11.10(81.97)	4	4.76(1.78,12.70)	4.75(8.39)	**0.008[a]**
	thrombocytopenia	88	9.89(7.99,12.24)	9.52(664.51)	33	3.87(2.74,5.46)	3.82(66.16)	**<0.001[b]**
	immune thrombocytopenia*	7	8.66(4.12,18.20)	8.64(39.89)	/	/	/	/
	haemolysis*	4	6.65(2.49,17.73)	6.64(13.91)	/	/	/	/
	haemolytic anaemia*&	4	4.90(1.84,13.08)	4.90(8.81)	4	5.25(1.97,14.01)	5.24(9.81)	0.458[a]
	lymphadenopathy*	10	3.36(1.81,6.26)	3.35(14.24)	/	/	/	/
	bone marrow failure*	6	2.98(1.34,6.64)	2.97(6.00)	/	/	/	/
	anamia	32	2.08(1.46,2.94)	2.06(16.50)	51	3.58(2.71,4.73)	3.52(89.87)	0.404[b]
	platelet count decreased#	108	12.46(10.26,15.12)	11.88(1067.16)	35	4.17(2.99,5.83)	4.12(79.76)	**<0.001[b]**

	febrile neutropenia	/	/	/	35	8.00(5.72,11.17)	7.88(203.40)	/
	neutropenia	/	/	/	49	4.59(3.46,6.10)	4.50(130.70)	/
	neutrophil count decreased[#]	/	/	/	59	20.12(15.52,26.08)	19.56(1018.48)	/
	white blood cell count decreased[#]	/	/	/	18	2.04(1.28,3.25)	2.03(8.46)	/
	disseminated intravascular coagulation	/	/	/	5	4.35(1.81,10.46)	4.34(9.72)	/
cardiac disorders	cardiac dysfunction	7	25.49(12.12, 53.63)	25.41(139.98)	6	23.38(10.47,52.18)	23.31(106.34)	0.150[a]
	cardiotoxicity	17	19.95(12.37, 32.18)	19.80(284.00)	/	/	/	/
	pericardial effusion*	10	5.05(2.71,9.41)	5.03(28.42)	/	/	/	/
	cardiac failure*	16	2.17(1.33, 3.55)	2.16(8.91)	/	/	/	/
	ejection fraction decreased[#]	18	12.26(7.70,19.50)	12.16(173.12)	22	16.09(10.57,24.51)	15.93(292.40)	0.150[b]
eye disorders	corneal disorder*	5	24.34(10.10,58.66)	24.29(89.15)	/	/	/	/
	dry eye	16	4.53(2.77,7.42)	4.51(40.27)	/	/	/	/
	blindness*	10	2.47(1.33,4.60)	2.46(7.30)	/	/	/	/
	keratitis[&]	/	/	/	5	21.95(9.11,52.89)	21.90(79.61)	/
gastrointestinal disorders	gingival bleeding*	9	8.24(4.28,15.86)	8.21(49.93)	/	/	/	/
	gastrointestinal toxicity	3	8.13(2.62,25.24)	8.12(12.26)	6	17.46(7.82,38.96)	17.41(76.89)	1.000[a]
	enteritis*	3	6.17(1.99,19.16)	6.16(8.32)	/	/	/	/
	ascites*[&]	12	4.84(2.75,8.54)	4.82(32.63)	37	16.23(11.72,22.48)	15.95(502.78)	0.147[b]
	varices oesophageal*	5	19.48(8.09,46.93)	19.44(69.73)	/	/	/	/
	colitis	/	/	/	9	3.33(1.73,6.41)	3.32(12.37)	/
	nausea	/	/	/	285	4.75(4.19,5.39)	4.22(719.78)	/
	vomiting	/	/	/	106	2.82(2.32,3.43)	2.73(116.38)	/
	ileus[&]	/	/	/	4	4.36(1.63,11.63)	4.35(7.25)	/
general disorders and administration site conditions	extravasation	11	27.83(15.37,50.42)	27.70(255.68)	/	/	/	/
	impaired healing*	6	2.51(1.12,5.59)	2.50(4.01)	/	/	/	/
	pyrexia	65	2.34(1.83,2.99)	2.30(46.88)	/	/	/	/
	chills	22	2.24(1.47,3.41)	2.23(13.71)	/	/	/	/
	fatigue	/	/	/	167	2.79(2.38,3.26)	2.64(173.35)	/
	mucosal inflammation	/	/	/	7	3.66(1.74,7.69)	3.65(10.95)	/
hepatobiliary disorders	blood bilirubin increased	22	9.05(5.95,13.79)	8.97(147.87)	15	6.59(3.96,10.95)	6.55(65.06)	**0.001[b]**
	nodular regenerative hyperplasia	17	225.09 (138.27,366.44)	223.32(3397.64)	/	/	/	/
	hepatic cirrhosis*	37	23.58(17.02,32.66)	23.19(760.65)	/	/	/	/
	non-cirrhotic portal hypertension	3	86.34(27.57, 270.43)	86.22(171.80)	/	/	/	/
	portal hypertension	23	85.34(56.40,129.13)	84.44(1785.01)	/	/	/	/
	hepatic fibrosis*	5	16.32(6.78,39.31)	16.29(57.10)	/	/	/	/
	hypertransaminasaemia	5	13.57(5.64,32.67)	13.54(46.09)	3	8.70(2.80,27.03)	8.69(13.43)	0.132[a]
	biliary obstruction*	3	11.52(3.71,35.78)	11.50(19.18)	/	/	/	/
	hyperbilirubinaemia*	8	9.71(4.85,19.46)	9.68(53.81)	/	/	/	/
	venoocclusive liver disease*	4	9.37(3.51,25.00)	9.35(22.03)	/	/	/	/

	hepatotoxicity	17	7.78(4.82,12.54)	7.72(92.84)	8	3.90(1.95,7.81)	3.89(14.41)	**＜0.001[b]**
	hepatic function abnormal	23	7.34(4.87,11.08)	7.27(118.26)	13	4.42(2.56,7.63)	4.40(30.86)	**＜0.001[b]**
	liver disorder	23	5.59(3.71,8.43)	5.54(81.16)	9	2.33(1.21,4.48)	2.32(5.51)	**＜0.001[b]**
	drug-induced liver injury	10	4.03(2.17,7.51)	4.02(19.75)	/	/	/	/
	cholecystitis*	4	3.74(1.40,9.99)	3.74(5.52)	/	/	/	/
	jaundice*	9	3.48(1.81,6.69)	3.46(13.42)	3	10.69(3.44,33.22)	10.68(17.50)	**0.005[b]**
	liver injury	6	3.13(1.40,6.97)	3.12(6.65)	/	/	/	/
	hepatic failure*	7	2.40(1.14,5.03)	2.39(4.36)	18	6.64(4.17,10.56)	6.59(79.82)	0.508[b]
	transaminases increased[#]	16	7.92(4.84, 12.96)	7.87(89.12)	/	/	/	/
	aspartate aminotransferase increased[#]	24	5.28(3.53, 7.90)	5.23(78.12)	/	/	/	/
	liver function test increased[#]	7	4.09(1.95,8.58)	4.08(13.32)	5	3.12(1.30,7.51)	3.12(5.23)	0.123[a]
	alanine aminotransferase increased[#]	21	3.93(2.56,6.04)	3.90(42.51)	/	/	/	/
	blood alkaline phosphatase increased[#]	8	3.65(1.82,7.32)	3.64(12.82)	/	/	/	/
	hepatic enzyme increased[#]	19	3.32(2.11,5.22)	3.30(28.25)	/	/	/	/
	gamma-glutamyltransferase increased[#]	6	3.29(1.48,7.34)	3.29(7.40)	/	/	/	/
	liver function test abnormal[#]	10	3.16(1.70,5.88)	3.15(12.60)	/	/	/	/
	cholangitis[&]	/	/	/	3	6.90(2.22,21.42)	6.89(9.78)	/
infections and infestations	encephalitis*	3	5.01(1.61,15.56)	5.01(6.03)	/	/	/	/
	rash pustular*	3	4.58(1.48,14.23)	4.58(5.19)	/	/	/	/
	cellulitis*	11	2.63(1.45,4.75)	2.62(9.46)	/	/	/	/
	pneumonia bacterial[&]	/	/	/	12	20.45(11.58,36.11)	20.33(200.99)	/
	pneumocystis jirovecii pneumonia[&]	/	/	/	19	20.03(12.74,31.49)	19.84(320.42)	/
	clostridium difficile colitis[&]	/	/	/	5	6.91(2.87,16.62)	6.89(19.62)	/
	neutropenic sepsis[&]	/	/	/	4	6.59(2.47,17.59)	6.58(13.74)	/
	septic shock[&]	/	/	/	16	5.32(3.25,8.69)	5.28(51.35)	/
	sepsis	/	/	/	20	2.34(1.51,3.63)	2.33(13.86)	/
injury,poisoning and procedural complications	infusion related reaction	18	5.21(3.28,8.29)	5.18(56.62)	/	/	/	/
	fracture[&]	/	/	/	7	3.07(1.46,6.46)	3.07(7.79)	/
investigations	ammonia increased*	4	8.73(3.27,23.31)	8.72(20.12)	/	/	/	/
	weight abnormal	/	/	/	4	20.10(7.52,53.69)	20.06(54.43)	/
	quality of life decreased[&]	/	/	/	5	3.53(1.47,8.50)	3.53(6.69)	/
metabolism and nutrition disorders	tumour lysis syndrome[&]	7	9.70(4.61,20.38)	9.67(46.01)	4	5.92(2.22,15.80)	5.91(11.77)	0.054[a]
	feeding disorder*	5	3.63(1.51,8.73)	3.62(7.04)	/	/	/	/

	hypercalcaemia*	8	8.31(4.15,16.64)	8.28(44.12)	/	/	/	/
	hypokalaemia*	10	2.80(1.51,5.22)	2.80(9.82)	/	/	/	/
	hyponatraemia*&	11	2.31(1.28,4.18)	2.31(6.89)	13	2.93(1.70,5.06)	2.92(14.55)	0.231[b]
	decreased appetite	/	/	/	61	3.34(2.59,4.31)	3.27(94.73)	/
musculoskeletal and connective tissue disorders	bone lesion*	3	9.77(3.15,30.36)	9.76(15.61)	/	/	/	/
	bone pain	12	2.28(1.29,4.02)	2.27(7.32)	/	/	/	/
	musculoskeletal pain	10	2.14(1.15,3.98)	2.13(4.95)	/	/	/	/
neoplasms benign,malinant and unspecified	focal nodular hyperplasia	3	48.38(15.51,150.92)	48.32(94.83)	/	/	/	/
nervous system disorders	peripheral sensory neuropathy	4	9.06(3.39,24.18)	9.04(21.10)	/	/	/	/
	neuropathy peripheral	71	8.97(7.08,11.37)	8.71(477.70)	/	/	/	/
	brain oedema*&	10	8.74(4.69,16.27)	8.70(60.60)	4	3.73(1.40,9.95)	3.72(5.48)	**0.008[a]**
	cerebral disorder*	6	8.65(3.88,19.28)	8.63(33.14)	/	/	/	/
	leukoencephalopathy*	3	7.79(2.51,24.20)	7.78(11.59)	/	/	/	/
	optic neuritis*	4	4.23(1.59,11.30)	4.23(6.89)	/	/	/	/
	epilepsy*	10	3.64(1.96,6.78)	3.63(16.50)	/	/	/	/
	neuralgia*	7	3.52(1.67,7.39)	3.51(10.17)	/	/	/	/
	cerebral haemorrhage*&	11	3.11(1.72,5.62)	3.09(13.58)	8	2.42(1.21,4.84)	2.41(5.27)	**0.030[b]**
	taste disorder&	/	/	/	6	5.07(2.27,11.29)	5.05(15.66)	/
	hypersomnia&	/	/	/	7	2.92(1.39,6.14)	2.92(7.01)	/
renal and urinary disorders	focal segmental glomerulosclerosis*	3	19.08(6.14,59.33)	19.06(34.73)	/	/	/	/
reproductive system and breast disorders	breast pain*	5	4.94(2.05,11.87)	4.93(11.96)	/	/	/	/
respiratory, thoracic and mediastinal disorders	pneumonitis	26	12.68(8.61,18.68)	12.54(264.30)	89	48.34(39.05,59.84)	46.24(3865.74)	**0.008[b]**
	epistaxis	57	8.77(6.74,11.41)	8.56(373.81)	/	/	/	/
	pulmonary fibrosis*&	11	8.77(6.74,11.41)	7.45(55.10)	6	4.36(1.96,9.72)	4.35(12.30)	**0.008[b]**
	interstitial lung disease	29	7.43(5.15,10.72)	7.35(152.72)	262	82.55(72.44,94.07)	71.88(18041.41)	**＜0.001[b]**
	pulmonary toxicity	4	6.53(2.45,17.42)	6.52(13.56)	18	31.83(19.99,50.69)	31.55(499.73)	0.111[b]
	pulmonary hypertension*	12	5.96(3.38,10.52)	5.93(44.41)	/	/	/	/
	pulmonary oedema*	9	2.31(1.20,4.44)	2.30(5.39)	/	/	/	/
	respiratory failure*	13	2.02(1.17,3.49)	2.01(5.68)	13	2.17(1.26,3.74)	2.16(6.97)	0.090[b]
	hepatopulmonary syndrome*	6	680.42(290.29,1594.81)	678.51(3016.32)	/	/	/	/
	lung disorder	17	4.01(2.49,6.46)	3.99(35.14)	21	5.32(3.46,8.18)	5.27(68.59)	0.171[b]
	pleural effusion*	17	3.33(2.07,5.37)	3.31(25.25)	30	6.35(4.43,9.10)	6.27(127.73)	0.781[b]
	lung opacity&	/	/	/	8	65.04(32.35,130.75)	64.78(435.55)	/
	pneumothorax&	/	/	/	5	3.75(1.56,9.03)	3.75(7.51)	/
	acute respiratory distress syndrome&	/	/	/	5	3.35(1.39,8.07)	3.35(6.05)	/
	hypoxia&	/	/	/	8	3.09(1.54,6.18)	3.08(9.26)	/
	acute respiratory failure	/	/	/	5	3.72(1.55,8.95)	3.71(7.38)	/
skin and subcutaneous	spider naevus*	8	522.14(252.12,1081.34)	520.19(3311.42)	/	/	/	/

tissue disorders	telangiectasia	8	110.21(54.65,222.28)	109.80(741.48)	/	/	/	/
	skin toxicity	5	11.26(4.68,27.12)	11.24(36.89)	/	/	/	/
	dermatitis acneiform*	3	6.25(2.01,19.39)	6.24(8.47)	/	/	/	/
	alopecia	/	/	/	65	3.89(3.04,4.98)	3.80(132.03)	/

*:Not mentioned in the T-DM1 instruction; &:Not mentioned in the T-DXd instruction; #:The PTs, although classified as "investigations" in SOC systems, were reassigned to a SOC system with similar physiological significance for the purpose of observation. a:Fisher's exact test; b:Chi-square test. Statistically significant values are marked in boldface.

Abbreviations: SOCs: System Organ Classes;N, the number of reports AE; PTs: Preferred Terms; ROR, reporting odds ratio; CI: confidence interval; PRR, proportional reporting ratio; $\chi 2$, chi-squared;
